# Electroacupuncture alleviates pain after total knee arthroplasty through regulating neuroplasticity: A resting‐state functional magnetic resonance imaging study

**DOI:** 10.1002/brb3.2913

**Published:** 2023-02-07

**Authors:** Bingxin Kang, Chi Zhao, Jie Ma, Haiqi Wang, Xiaoli Gu, Hui Xu, Sheng Zhong, Chenxin Gao, Xirui Xu, Xinyu A, Jun Xie, Mengmeng Du, Jun Shen, Lianbo Xiao

**Affiliations:** ^1^ Department of Rehabilitation centers The First Affiliated Hospital of Henan University of Chinese Medicine Zhengzhou China; ^2^ Acupuncture Tuina Institute Henan University of Chinese Medicine Zhengzhou China; ^3^ Center of Rehabilitation Medicine Yueyang Hospital of Integrated Traditional Chinese and Western Medicine, Shanghai University of Traditional Chinese Medicine Shanghai China; ^4^ Department of Orthopaedics Shanghai Guanghua Hospital of Integrative Chinese and Western Medicine Shanghai China; ^5^ Arthritis Institute of Integrated Traditional Chinese and Western Medicine Shanghai University of Traditional Chinese Medicine Shanghai China; ^6^ Depart of Peripheral vascular The First Affiliated Hospital of Henan University of Chinese Medicine Zhengzhou China

**Keywords:** acute pain, electroacupuncture, resting‐state functional magnetic resonance imaging, total knee arthroplasty

## Abstract

**Introduction:**

We aimed to evaluate the efficacy of electroacupuncture in relieving acute pain after total knee arthroplasty (TKA) and related mechanism.

**Methods:**

In this randomized, single‐blind, and sham‐acupuncture controlled study. Forty patients with postoperative acute pain were recruited and randomly divided electroacupuncture group (*n* = 20) and sham‐acupuncture group (*n* = 20) from November 2020 to October 2021. All patients received electroacupuncture or sham‐acupuncture for 5 days after TKA. Their brain regions were scanned with resting‐state functional magnetic resonance imaging before and after intervention. Pain was scaled. Another 40 matched healthy controls underwent scanning once. The amplitude of low‐frequency fluctuation (ALFF) values was compared. Pearson's correlation analysis was utilized to explore the correlation of ALFF with clinical variables in patients after intervention.

**Results:**

Compared with the HCs, patients with acute pain following TKA had significantly decreased ALFF value in right middle frontal gyrus, right supplementary motor area, bilateral precuneus, right calcarine fissure and surrounding cortex, and left triangular part of inferior frontal gyrus (false discovery rate corrected *p* < .05). Patients had higher ALFF value in bilateral precuneus, right cuneus, right angular gyrus, bilateral middle occipital gyrus, and left middle temporal gyrus after electroacupuncture (AlphaSim corrected *p* < .01). Correlation analysis revealed that the change (postoperative day 7 to postoperative day 3) of ALFF in bilateral precuneus were negatively correlated with the change of NRS scores (*r* = −0.706; *p* = .002; 95% *CI* = −0.890 to −0.323) in EA group.

**Conclusions:**

The functional activities of related brain regions decreased in patients with acute pain after TKA. The enhancement of the functional activity of precuneus may be the neurobiological mechanism of electroacupuncture in treating pain following TKA.

## 1 INTRODUCTION

Total knee arthroplasty (TKA) can effectively treat end‐stage knee osteoarthritis (KOA) by reducing pain and helping patients to resume daily life activities. Acute postoperative pain after TKA prolongs rehabilitation duration, weakens the therapeutic effect, and thereby decreases the patient's quality of life. Intense postoperative pain is associated with chronic pain, and it may also prolong rehabilitation duration, weaken the therapeutic effect of TKA, and thereby decrease the patient's quality of life (Coppes et al., 2020; Hsia et al., 2018). The American Society of Anesthesiologists has recommended that multimodal pain management (administration of two or more drugs via the same route or different routes) should be implemented whenever possible to maximize the analgesia effect while minimize the potential adverse effects and reduce the consumption of opioids (American Management, 2012). But the incidence of moderate to severe pain following TKA is still as high as 58%, indicating that postoperative pain management is far from satisfactory (Summers et al., 2020). Conventional analgesia medicine could increase the risk of nausea, vomiting and other digestive system side effects, abnormal liver/ renal functions, and inhibit bone formation and healing (Zhao & Davis, 2019). Thus, effective and safe nonpharmacologic interventions for analgesia are necessary.

Acupuncture, a methodology of treating human diseases in China with a history of more than 3000 years, has been applied to various pain disorders, such as migraine (Tu et al., 2020), KOA(Kong et al., 2018), low back pain (Yu et al., 2020), fibromyalgia pain (Mawla et al., 2021), neck pain (Li et al., 2020), and cancer pain (Liang et al., 2020; Yang et al., 2021). Increasing evidence has proved the safety and effectiveness of acupuncture as an analgesic treatment and its advantage in decreasing the need for opioids (Michaelides & Zis, 2019). Neuroimaging studies have shown that acupuncture can achieve analgesic effect by restoring the pain processing, regulating pain perception, improving abnormal structure, and functional activities of patients (Tian et al., 2021; Wen et al., 2021). Electroacupuncture (EA), which combines the traditional acupuncture theory with electrical stimulation, has standardized intensity, frequency, duration and other parameters, making it more suitable for scientific studies than traditional manual acupuncture. EA has been found to relieve pain and reduce opioid consumption following TKA (Tedesco et al., 2017). The EA's analgesic effect on neuropathic pain may rely on the activation the brain functional connectivity between bilateral hemispheres and the sensorimotor cortex (Hou et al., 2020). The descending pain modulation systems, including the anterior cingulated cortex, the periaqueductal gray, and the rostral ventromedial medulla, play an important role in the analgesic effect of EA (Chen et al., 2020). Although the central integration and plasticity play a critical role in the analgesic mechanism of acupuncture (Xiao et al., 2018), the underlying mechanism of EA in regulating the brain central system to relieve acute pain following TKA is largely unknown.

Resting‐state functional magnetic resonance imaging (rs‐fMRI), which gauges fluctuations in the blood oxygen level‐dependent signal, is widely used to explore the neural mechanisms, assess the efficacy of acupuncture treatment, and it is also commonly used for pain research. The amplitude of low‐frequency fluctuation (ALFF), which depicts the intensity of regional spontaneous neuronal activities, strikes a good balance between test‐retest reliability and replicability (Chen et al., 2018). ALFF reflects the blood oxygenation level‐dependent (BOLD) signal fluctuations within the gray matter and the local properties of spontaneous neuronal activity. The enhancement of ALFF shows that the excitability of brain area is activated, and the BOLD signal deviated from the baseline. The weakening of ALFF indicates that neurons are inhibited and their activities are decreased. ALFF can be represented as the square root of the power spectrum in low‐frequency range (0.01–0.08 Hz), which measures evaluate the brain's pathophysiological state by computing the regional intensity of spontaneous fluctuation in BOLD signal at rest (Zang et al., 2007). Studies no various diseases such as heroin addicts (Luo et al., 2020), trigeminal neuralgia(Ge et al., 2022), migraine (Chen et al., 2021), cervical discogenic pain (Ma et al., 2020), chronic low back pain(Zhang et al., 2019), fibromyalgia (Katherine et al., 2019), suggest that ALFF, as a reliable indicator of regional spontaneous neural activity in resting‐state, can be widely used in pain disease studies.

In this study, we used rs‐fMRI to explore the brain central mechanism of EA in treating acute pain following TKA. To our knowledge, few neuroimaging research has focused on this area. We speculated that (1) the ALFF patterns in patients with acute postoperative pain might be abnormal, and (2) EA could treat acute pain after TKA by regulating the functional activities of specific brain regions.

## 2 MATERIALS AND METHODS

### 2.1 Standard protocol approvals, registration, and consents

The study took place at Shanghai Guanghua Hospital of Integrated Traditional Chinese and Western Medicine from November 1, 2020 to October 31, 2021. The study protocol was preregistered with ClinicalTrials.gov (No.ChiCRT2000033778, June 14, 2020, https://www.chictr.org.cn/edit.aspx?pid=54096&htm=4). All study protocols were approved by the Ethics Committee of Shanghai Guanghua Hospital of Integrated Traditional Chinese Medicine and Western Medicine (2020‐K‐44), and all participants provided written informed consent in accordance with the Declaration of Helsinki.

### 2.2 Study participants

Enrolled were KOA patients having undergone primary unilateral TKA. The inclusion criteria included (1) aged 60–80 years; (2) right‐handed; (3) a pain intensity score of 5 or higher on a 0–10 Numerical Rating Scale (NRS); (4) a score > 24 on the Minimum Mental State Examination. The excluded criteria included (1) a history of neurological and psychiatric disorders, or head trauma with loss of consciousness; (2) serious renal, cardiovascular, respiratory or other organic diseases; (3) any contraindications to rs‐fMRI scanning (such as defibrillator, cardiac pacemaker, metal stents or electronic implant, intraocular mental foreign body, claustrophobia, and hyperpyrexia); (4) unwillingness to sign the consent form; (5) having received acupuncture/EA in the past three months.

The sex‐ and age‐matched healthy controls (HCs) without any illness‐induced pain sensation or psychological diseases were also recruited.

### 2.3 Experimental design and sample size

A participant‐blinded, randomized and sham‐acupuncture (SA) controlled clinical trial was applied. Previous studies have shown that 15 participants should be included in each group to ensure stable statistical effect for brain fMRI analysis (Qiu et al., 2016; Szucs & Ioannidis, 2020). In this study, rs‐fMRI was conducted to explore the cerebral mechanism rather than observe the clinical efficacy. The participants were randomly assigned to either EA or SA group (*n* = 20 each) for rs‐fMRI scanning.

The study lasted for 5 days: from postoperative day (POD) 3 to POD 7. Patients were instructed to completed postoperative pain diary by documenting the onset time, pain intensity (measure by NRS score), rescue medication use, and rs‐fMRI scanning, which collected at POD 3 and POD 7.

### 2.4 Masking and intervention

The group allocation information was sealed an envelope and given to the acupuncturist. The surgeons, principal investigators, study staff, data analysts, and the participants were blinded to grouping. Using the sham electroacupuncture design in the previous literature(Liu et al., 2017), adhesive pads were applied to both groups, thus the blunt‐tipped placebo needles with a similar appearance to conventional needles provided participant‐blinding effects but no skin penetration. Besides, the SA included a connecting cord with a broken inner wire with no actual current output. Participants in the EA group received acupuncture. Acupoint locations were selected based on the standardized acupuncture protocol for TKA (Zhong et al., 2019). Four acupoints at the surgical limb, including Futu (Stomach 32, ST32), Zusanli (Stomach 36, ST36), Yinlingquan (Spleen 9, SP9), and Yanglingquan (Gall Bladder 34, GB34), were selected. After skin disinfection, sterile adhesive pads were placed on ST32, ST36, SP9, and GB34, and the sterile disposable acupuncture needles (0.25 mm diameter, 40 mm length, stainless steel) were inserted through the adhesive pads approximately 20–35 mm in to the skin, depending on the thickness of the local tissues. The inserted needles were moved until the “Deqi” sensation (a composite of sensations including soreness, numbness, distention, heaviness) was achieved without causing a sharp pain. Then the needles, which were connected to an EA machine (SDZ‐II, Huatuo, Suzhou, China), with a pair of electrodes connecting GB 34 with ST 32, and another pair of electrodes connecting ST36 to SP9 (Zhong et al., 2019), put through a continuous wave of 2 Hz and 1 to 5 mA current intensity into the skin. The electric stimulation was enhanced gradually to the highest tolerable level for the patient without causing pain, and retained for 20 min.

In SA group, noninserted sham needles were applied to the same acupoints as in EA group. The electrodes were attached to these needles with, the same treatment setting as in the EA group. The EA device was turned on, but the electrodes were not inserted into an active port on the device, and no skin penetration or needle manipulation was achieved for “De qi.” Intervention was not performed on HCs. All the patients included in the final analysis completed 5 treatment sessions during the 5‐day treatment.

### 2.5 Clinical assessment

Nonsteroidal anti‐inflammatory drug was used for analgesia, and supplementary dosage could be administered if needed. No additional analgesic medication was asked for in both EA and SA groups throughout the study. All patients underwent rs‐fMRI scan twice. The demographic information and clinical scale data (dependent variables) at POD 3 (preintervention) and POD 7 (postintervention) were analyzed. The primary outcome was mean reduction in pain intensity represented by the NRS score of patients. To obtain the NRS score, the patients were asked to circle a number ranging from 0 (no pain) to 10 (the most intense pain imaginable) that best fit their current level of pain. The reduction in Zung Self‐Rating Depression Scale (SDS) score was calculated as the secondary outcome. SDS was used to evaluate the subjectively reported depression by the patients. The scale consisted of 20 items and each item was divided into 4 levels according the frequency of symptoms, which is suitable for adults with depression. For the SDS score, patients scored on the scale according to their emotional state.

### 2.6 Rs‐fMRI data acquisition

The rs‐fMRI data were acquired using a Clinical 1.5 Tesla whole body MR imager (United Imaging, Shanghai, China). Although our previous clinical trial (Kang et al., 2022) had verified the reliability of 1.5 Tesla data, we repeatedly tested the stability of the machine before the start of this study to ensure the reliability of the data. A head‐hugger and earplugs were used to minimize noise and head movement during scanning. Participants were instructed to keep their eyes closed, relax, stay awake, and not think about anything in particular. Rs‐fMRI images were obtained by a rapid‐gradient echo‐planar imaging sequence with the following setting: repetition time, 3000 ms; echo time, 30 ms; flip angle, 90°; field of view, 225 × 225 mm^2^; acquired matrix, 64 × 64 matrices; 43 slices with a thickness of 3.5 mm; voxel size, 3.52 × 3.52 × 3.52 mm^3^; bandwidth, 2250 Hz/pixel. The scanning duration was 12 min and 13 s. In response to a questionnaire after the scan, all participants stated that they had not fallen asleep.

### 2.7 Data processing and analyzing

#### 2.7.1 Statistical analysis

Clinical outcomes were analyzed using a statistical package for windows version 25 (SPSS, IBM Inc., Chicago, IL). A single‐factor ANOVA/two‐sample *t*‐test and a *χ* ^2^ test was applied to compare the baseline characteristics of the participants. The NRS and SDS scores were compared using a linear mixed model with group, time allocation and interaction between the two as fixed effect. The mean (standard deviation, SD) was presented for continuous variables; while frequency was used for categorical data with corresponding *p* and *t*/*F* values. A two‐side test was applied, with a confidence interval of 95% and *p* < .05 indicating statistically significant.

#### 2.7.2 ALFF analysis

The data was processed by the Data Processing Assistant for rs‐fMRI (Restplus) based on Statistical Parametric Mapping 12 (SPM 12, http://www.fil.ion.ucl.ac.uk/spm/software/spm12/) and run using MATLAB R2014. Before analysis, we performed left and right head flip in patients underwent left TKA, thereby lateralizing the reflection area to the same hemisphere in all patients. The processing included (Xu et al., 2020) (1) discarding the first 10 volumes; (2) slice‐time correction; (3) head movement correction (translational or rotational motion parameters < 3 mm or 3°); (4) spatial normalization to the standard template and resampling to a 3×3×3 mm voxel size; (5) spatial smoothing with an 6 mm full width at half maximum (FWHM) kernel; (6) linear trend removal; (7) regression of nuisance covariates (including the white matter, cerebral spinal fluid, and the Firston 24 head motion parameters) (Yan et al., 2013)

The blood oxygen level‐dependent time series for each voxel was converted to the frequency domain with fast Fourier transform. The square root of the power spectrum was computed and averaged across the specified frequency range (0.01–0.08 Hz) at each voxel. The averaged square root was used as the ALFF, which was transformed by Fisher's *z* transformation for subsequent group‐level analysis.

To investigate the alternations in HCs and patients in POD 3, we used false discovery rates (FDR) correction for multiple comparison (voxel‐*p* < .05, cluster‐*p* < .05). For patient group‐level analyses, we used AlphaSim correction for multiple comparison (voxel‐*p* < .01, cluster‐*p* < .01). The ALFF was analyzed by two‐sample *t*‐test and paired *t*‐test using SPM 12 software.

#### 2.7.3 Correlation analysis

The EA‐stimulation difference (EA_ POD 7 vs. POD 3) and group difference (HCs vs. patients at POD 3) were chosen as the regions of interest (ROI). The mean values in the ROI between the two groups were analyzed using the receiver operating characteristic curves. Pearson's correlation coefficients were calculated to indicate the relationship between the mean ALFF values in ROI in patients, where *p* < .05 was considered as a significant difference.

## 3 RESULTS

### 3.1 Baseline characteristics

**FIGURE 1:**
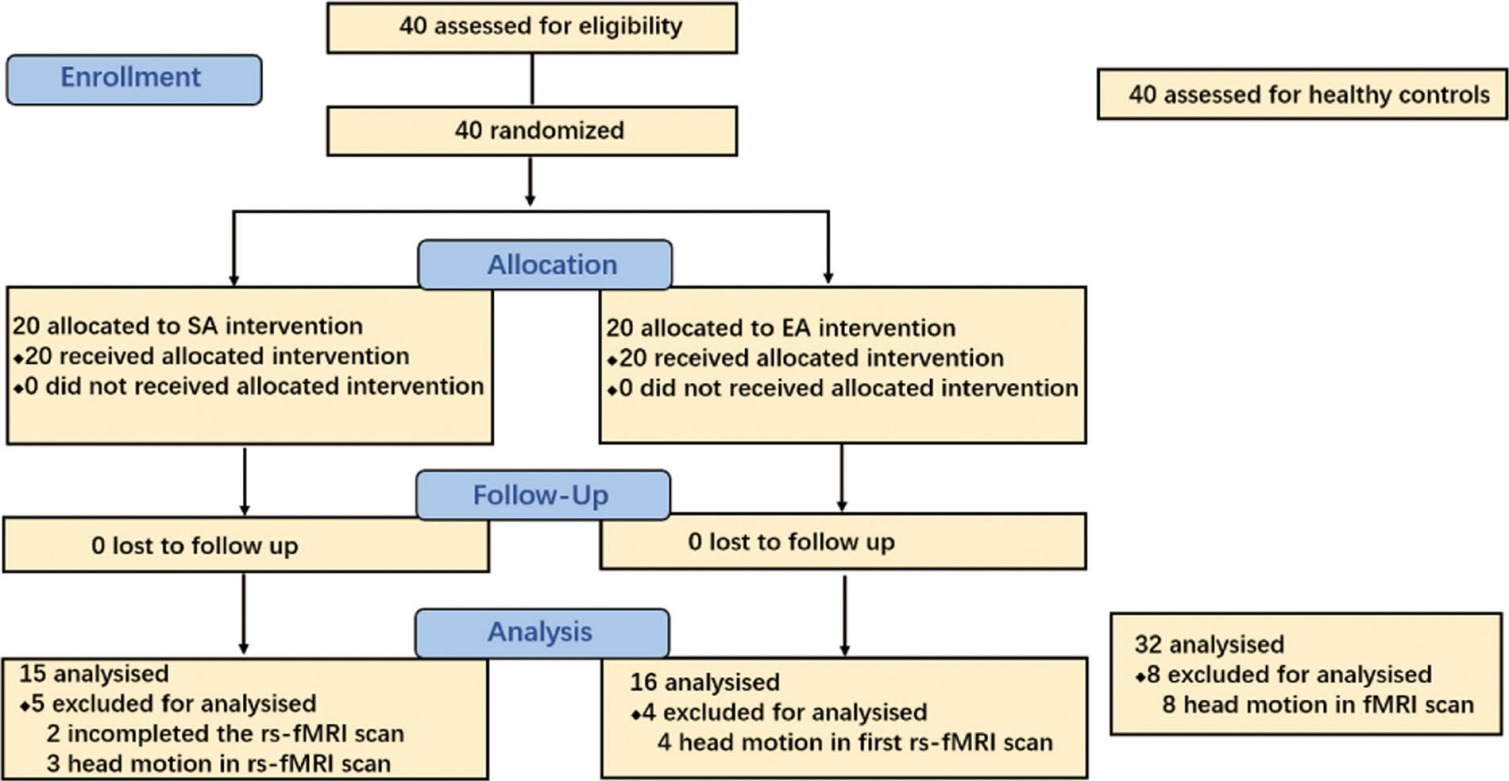
Folw chart of screening, randomization and intervention. SA, sham‐acupuncture; EA, electroacupuncture; rs‐fMRI, resting‐state functional magnetic resonance imaging.

Totally, 38 patients (20 in EA group, 18 in SA group) completed the two rs‐fMRI scans (baseline and after 5‐day treatment). Forty HCs completed one rs‐fMRI scan. Due to excessive head movement (>3 mm) in scan, 7 patients and 8 HCs were excluded (Figure 1). No significant differences in age, gender, and body mass index were observed between patients and HCs. Table 1 shows that there was no difference between in the EA and SA group in pain intensity and SDS at baseline (age, gender, and body mass index).

**TABLE 1:** Demographic and clinical characteristics

Characteristic	HCs	Patients before intervention		*F/χ^2^*	*p* Value
		EA group	SA group		
Number of cases, *n*	32	16	15	—	—
Age (years)	68.8 (4.4)	71.4 (6.1)	69.4 (5.0)	1.373	.261
Gender (M/F), *n*	10/21	2/14	2/13	3.021	.221
BMI (kg/m^2^)	24.3 (2.0)	25.0 (1.9)	25.7 (1.7)	2.796	.069

Results are presented as mean (SD).

EA, electroacupuncture; SA, sham‐acupuncture; HCs, healthy controls; M, meal; F, female; BMI, body mass index.

### 3.2 Clinical outcomes

Table 2 summarizes the clinical outcome and statistics. Significant time × group interaction effects were found in pain intensity (mean reduction in NRS [*F* = 15.634, *p* < .001]) and emotional state (mean reduction in SDS [*F* = 4.827, *p* = .036]).

**TABLE 2:** Clinical outcome measurements in the EA and SA groups

	EA group, *n* = 16			SA group, *n* = 15			Interaction effect	
	Pretreatment mean (SD)	Posttreatment mean (SD)	Post‐pre (95% *CI)*	Pretreatment mean (SD)	Posttreatment mean (SD)	Post‐pre (95% *CI*)		Effect size
NRS	6.0 (0.6)	3.5 (0.6)	−2.2 (−2.4, −2.5)	6.1 (0.6)	4.7 (0.6)	−1.4 (−1.4, −1.5)	*p* < .001	0.842
SDS	46.1 (3.9)	39.1 (2.9)	−7.8 (−6.8, −7.1)	46.6 (2.8)	42.7 (3.3)	−3.9 (−3.8, −4.0)	*p* = .036	0.575

EA, electroacupuncture; SA, sham‐acupuncture; NRS, numerical rating scale; SDS, Self‐Rating Depression Scale.

### 3.3 ALFF analysis

**FIGURE 2:**
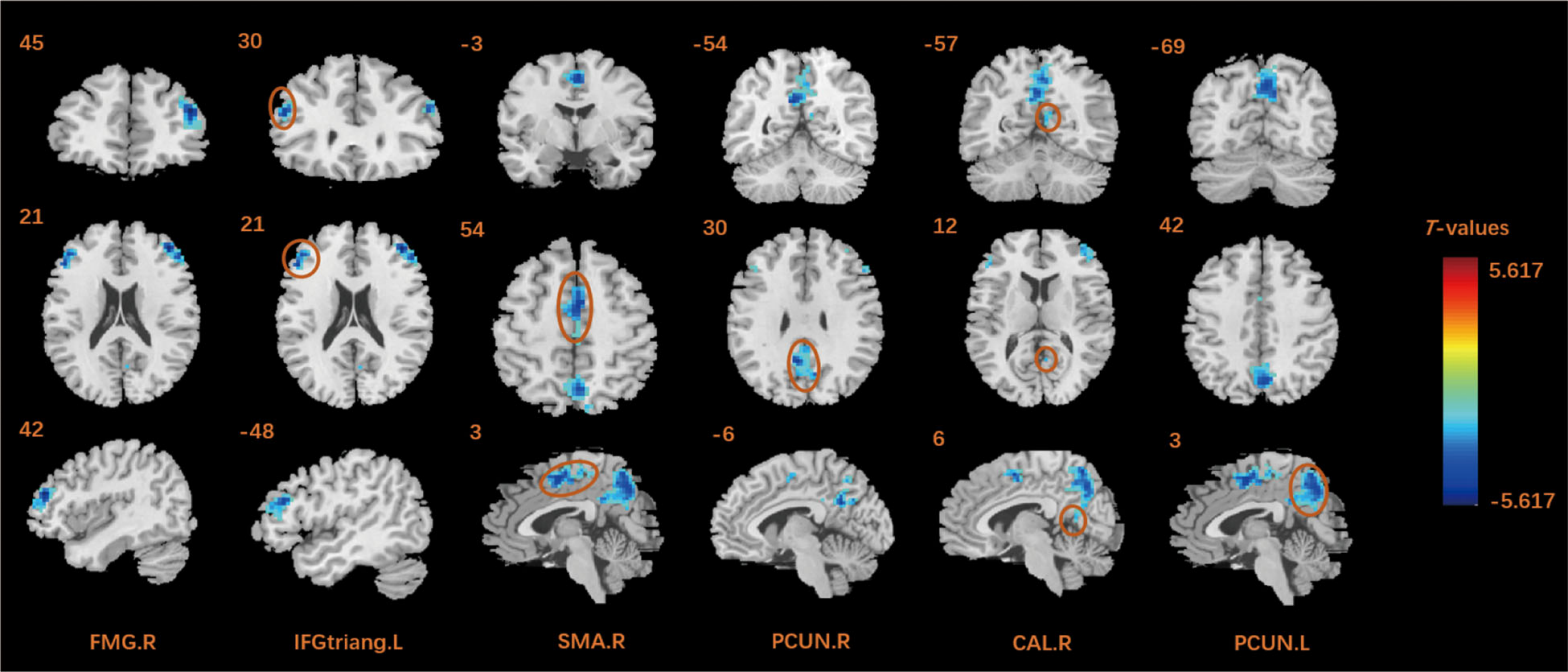
Comparisons of ALFF between patients in postoperative day 3 and healthy controls. Blue represents the area with decreased ALFF value. ALFF, amplitude of low‐frequency fluctuation; FMG, middle frontal gyrus; IFGtriang, triangular part of inferior frontal gyrus; SMA, supplementary motor area; PCUN, precuneus; CLA, calcarine fissure and surrounding cortex; L, left; R, right.

Compared with HCs, all patients at POD 3 had significantly lower ALFF in the right middle frontal gyrus (MFG), right supplementary motor area (SMA), bilateral precuneus, right calcarine fissure and surrounding cortex (CAL), and left triangular part of inferior frontal gyrus (IFGtriang) (Table 3, Figure 2).

**TABLE 3:** Comparisons of ALFF values between patients at POD 3 and HCs

			MNI coordinates			
Cluster regions	Cluster size	L/R	*X*	*Y*	*Z*	*t* Value
Middle frontal gyrus	152	R	42	45	21	−5.617
Supplementary motor area		R	3	−3	54	−5.225
			3	−24	60	−4.074
Precuneus	446	L	−6	−54	30	−5.136
		R	3	−69	42	−4.942
Calcarine fissure and surrounding cortex		R	−6	−54	30	−5.136
Triangular part of inferior frontal gyrus	86	L	−48	30	21	−4.979

Coordinates (*X*, *Y*, *Z*) refer to the peak MNI coordinates of brain regions with peak intensity. The resulting statistical map uses false discovery rates correction for multiple comparisons analysis (voxel‐*p* < .05, cluster‐*p* < .05, cluster size > 63).

ALFF, amplitude of low‐frequency fluctuation; MNI, Montreal Neurological Institute; POD3, postoperative day 3; HCs, healthy controls; L, left; R, right.

**FIGURE 3:**
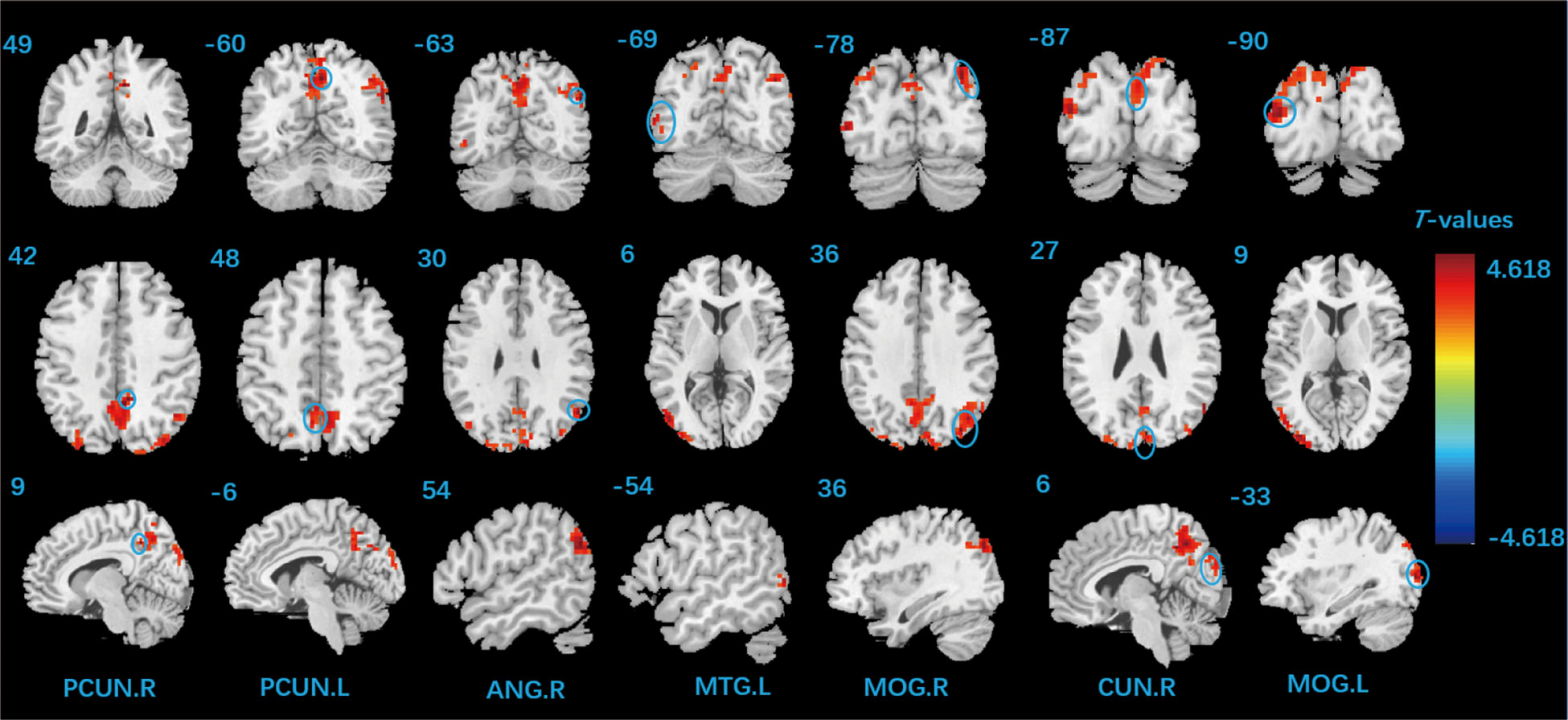
Comparisons of ALFF before and after treatment in electroacupuncture group. Red represents the area with increased ALFF value. ALFF, amplitude of low‐frequency fluctuation; PCUN, precuneus; ANG, angular gyrus; MTG, left middle temporal gyrus; MOG, middle occipital gyrus; CUN, cuneus; L, left; R, right.

In EA group, the patients showed significantly higher ALFF at POD 7 in bilateral precuneus, right cuneus, right angular gyrus, bilateral middle occipital gyrus (MOG), and left middle temporal gyrus (MTG), compared with those at POD 3 (Table 4, Figure 3). In SA group, no significant differences in ALFF were observed between POD 3 and POD 7.

**TABLE 4:** Comparison of the ALFF between POD 7 and POD 3 in EA group

			MNI coordinates			
Cluster regions	Cluster size	L/R	*X*	*Y*	*Z*	*t* Value
Precuneus	288	R	9	−48	42	4.618
		L	−18	−78	54	3.857
Cuneus		R	6	−87	27	3.521
Angular gyrus	119	R	54	−63	30	4.451
Middle occipital gyrus	119	R	36	−78	36	4.364
	185	L	−33	−90	9	4.314
			−42	−75	33	3.647
Middle temporal gyrus		L	−54	−69	6	3.938

Coordinates (*X*, *Y*, *Z*) refer to the peak MNI coordinates of brain regions with peak intensity. The resulting statistical map uses AlphaSim correction for multiple comparisons analysis (voxel‐*p* < .01, cluster‐*p* < .01, cluster size > 107).

ALFF, amplitude of low‐frequency fluctuation; MNI, Montreal Neurological Institute; POD3, postoperative day 3; POD7, postoperative day 7; L, left; R, right.

### 3.4 Correlation between ALFF and pain intensity after intervention

**FIGURE 4:**
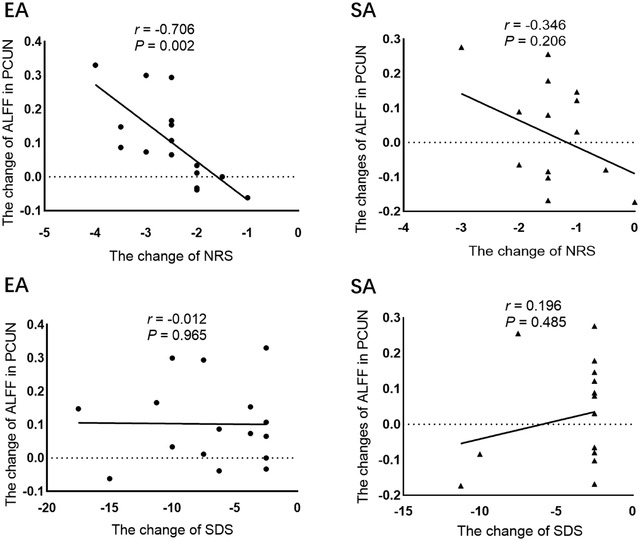
Correlation between the change of ALFF in precuneus with the change of NRS scores and SDS in patients. The change (POD7–POD3) of ALFF in precuneus was negatively correlated with the change of NRS scores and not significantly correlated with the change of SDS in EA group. There was no significant difference between the changed ALFF values of precuneus with the changed NRS scores and SDS. ALFF, amplitude of low‐frequency fluctuation; PCUN, precuneus; NRS, numerical rating scale; SDS, Self‐Rating Depression Scale.

The EA‐stimulation difference (EA_ POD 7 vs. POD 3) and group difference (HCs vs. patients at POD 3) as shown by ALFF overlapped in bilateral precuneus, which was chosen as the ROI. Correlation analysis revealed that the change (POD 7–POD 3) in ALFF of bilateral precuneus were negatively correlated with the change of NRS scores (*r* = −0.706; *p* = .002; 95% *CI* = −0.890 to −0.323) and not significantly correlated with the change of SDS score (*r* = −0.012; *p* = .965; 95% *CI* = −0.505 – −0.487) in EA group. There was no significant difference between the changed (POD7–POD3) ALFF values of bilateral precuneus correlated with the change of NRS (*r* = −0.346; *p* = .206; 95% *CI* = −0.729 – −0.201) and SDS (*r* = 0.196; *p* = .485; 95% *CI* = −0.352 – −0.644), respectively (Figure 4).

### 3.5 Complications and adverse events

All incisions were healed by the first intention. No skin ulcer, hematoma, infection, liver or kidney injury, or other adverse events was observed.

## 4 DISCUSSION

The transmission of pain in the central nervous system is highly complicated and involves multiple brain regions (Kim et al., 2021; Qi et al., 2018). In this study, we investigated the underlying neurobiology of postoperative acute pain and the modulatory effect of EA on acute pain after TKA.

We found that patients had lower ALFF in right MFG, right SMA, bilateral precuneus, right CAL, and left IFGtriang following TKA (preintervention), compared with HCs without any pain symptoms. MFG and IFGtriang in the prefrontal cortex can modulate pain perception (Morton et al., 2016). The MFG activity is implicated in pain stimulation and modulation, perception of negative emotions, and cognitive evaluation (Boissoneault et al., 2020). Deactivating the left IFGtriang is associated with response inhibition, as well cognitive and emotional dysregulation (Liu et al., 2020). Precuneus, a cortical element in the medial surface, is involved in attention, spatial integration, self‐awareness, psychological activities, social cognition, and emotional processing (Pereira‐Pedro & Bruner, 2016). CAL, surrounded by the primary visual cortex, is related to cognitive function (Yang et al., 2020). Although all participants in this study showed normal cognitive function, the decreased functional activities in the above brain regions may lead to the experience of intense pain in patients, further reducing their brain activities and weakening cognitive function (Huang et al., 2015). We speculate that patients with pain sensitivity after TKA may have a higher risk of pain‐induced cognitive impairment, which is associated with the changed functional brain activities. The SMA in the frontal cortex can delimit the motor from the prefrontal cortical regions and link cognition to action in normal behaviors, involving movement preparation, short‐term retention of pain dynamics, and other cognitive and motor‐related processes (Khoshnejad et al., 2017). The decreased functional activities of SMA may be related to the knee motor dysfunction. In KOA patients, the pathological changes influenced the spatial patterns of intrinsic brain activity, leading to altered brain function and structure (Kang et al., 2022). The deactivation of these regions involved in pain regulation may indicate inadequate inhibition or increased facilitation of sensory information, which is associated with functional impairment in pain processing, thus contributing to acute pain after TKA.

This preliminary clinical trial has shown that EA reduced pain intensity and depression tendency after TKA. The analgesia effect of EA may be achieved through regulating the plasticity of brain functional activities, while SA cannot, exert such an effect. And this explains why the patients in EA group had higher ALFF in right cuneus, right angular gyrus, bilateral MOG, right MTG, and bilateral precuneus at POD 7 than at POD 3.

The cuneus integrates somatosensory input with other sensory stimuli and cognitive processes, involving the emotion dimension of pain (Price, 2000). The angular gyrus is implicated in cognitive functions, including attention and spatial cognition, default mode network, and social cognition (Seghier, 2013). Factors associated with cognition are likely to modulate pain perception. The surgical injury triggers a myriad of responses in the pain matrix, from sensitization of central pain pathways to anxiety and depression (Small & Laycock, 2020). Emotion affects pain perception, and patients experiencing severe acute pain are more likely to develop a negative emotion, which in turn increases pain perception, thus forming a vicious circle (Michaelides & Zis, 2019). Decreased ALFF of left MOG is observed in depressive patients (Teng et al., 2018). Temporal cortex participates in pain perception and modulation, as confirmed in human neuropathic states, and it is also closely related to emotional control and sensory process modulation, and pain sensation duration (Khoshnejad et al., 2017). The SDS score in EA group showed the patients receiving EA had a lower tendency to develop depression, which can be explained by the reduced pain severity and negative emotions after EA, and the resultant increase of the functional activities in the relevant brain regions. Another explanation is EA stimulation directly activates the relevant brain areas to regulate the adverse emotions and reduce the perception of acute pain. Pain relief could be evidenced by the reduction of NRS score in the EA group, compared with the SA group, although both received a conventional analgesic agent during the whole study. Changes of these brain functional activities indicate that EA relieves the pain by increasing the activities of the brain regions associated with the pain modulation, and thus changing the pain perception from nociceptive receptors to the pain matrix.

The EA‐stimulation (EA_POD 7 vs. POD 3) and group (HC vs. POD 3) difference as shown by ALFF overlapped in precuneus. The nucleus cuneiformist is an inhibitory region in the descending pathway, and the hypoactivity in this region indicates reduced inhibition of the pain response in patients, which produces a higher pain score (Schwedt et al., 2015). Precuneus, a key region in the neuronal network for continuous information gathering and assessment of self‐relevant sensations, mediates intrinsic activities throughout the default model network (Goffaux et al., 2014). The ALFF in precuneus decreased when acute pain is evoked in chronic low back pain patients (Zhang et al., 2019). When receiving pain stimuli, precuneus presents deactivation, and pain sensitivity is negatively correlated with the activity in the precuneus (Goffaux et al., 2014). The correlation analysis between the reduction of NRS score and the ALFF change of bilateral precuneus in the present study, we found that the activated changes in precuneus may play a crucial role in modulating postoperative pain perception. And we speculate that EA increases the functional activity of precuneus to reduce the postoperative pain sensitivity and perception, which may be a central mechanism of EA in alleviating acute pain after TKA.

The pain intensity of patients in the SA group was also gradually eased over time after TKA. This may be attributed to the fact, the patients tended to limit their limb movement on the operative side to reduce the pain sensation due to the poor analgesic effect of SA. No changes (POD 7 vs. POD 3) in brain regions related to pain regulation were found on rs‐fMRI in SA group. The different changes of brain regions in the two groups indicated the central mechanisms of EA and SA were different. Part of the therapeutic effect of SA may be produced by the physiological effects on the skin during the stimulation. It should be noted that the AlphaSim correction was applied to explore the mechanism of EA analgesia in our study. Although the precuneus functional activities in EA group were closer to those HCs, the finding did not survive the more stringent FDR correction methods, and the AlphaSim correction method is relatively loose; therefore, further studies are needed.

Taken together, we propose that EA relieves acute pain after TKA by increasing right cuneus, right angular gyrus, bilateral MOG, left MTG, and precuneus functional activity. The increased ALFF in pain‐related brain regions may exert beneficial impacts on pain‐regulating functions in patients after TKA. Besides, the EA‐stimulation and group difference overlapped in precuneus, suggesting that precuneus functional activities increase with the decreased pain intensity, which is pivotal to pain processing and regulation. Our research confirms the abnormal decrease of the precuneus functional activity can be reversed by EA, but the results need further validation.

Our study has several limitations. (1) The small sample size of the study may increase the likelihood of a false positive error. Ours are preliminary findings, which lack prior power calculations, and thus should to be verified by studies with larger sample sizes. (2) This study only verifies the EA effect at the group level, and the specificity of EA analgesia is not fully explained. Future studies are needed to confirm the immediate effect of EA and validate our findings. (3) Due to excessive head movements, quite a few participants were excluded; but it should be emphasized that the exclusion of the images exerts no impact on treatment response. (4) Due to some practical difficulties and the limitations of experimental conditions, we failed to enroll an untreated group to investigate the effect of elapsed time on pain management after TKA. Hopefully, we will overcome the disadvantages in the subsequent work.

## 5 CONCLUSION

In conclusion, the functional activities in the right MFG, right SMA, bilateral precuneus, right CAL, and left IFGtriang in patients with acute pain after TKA decreased. EA on four acupoints (Futu, Zusanli, Yinglingquan, and Yanglingquan) can increase the functional activities of right cuneus, right angular gyrus, MOG, left MTG, and precuneus. The functional activity of the precuneus is a biomarker of pain after TKA. Enhancement of functional activity of precuneus may be the neurobiological mechanism of EA in treating acute pain following TKA.

## AUTHOR CONTRIBUTIONS

BXK, CZ, and JM conceived the study; BXK drafted the study; HQW, XLG, HX, SZ, CXG, and XRX recruited the participants. XYA and JX collected clinical data. MMD and CZ were responsible for statistical analyses and tables. LBX and JS have primary responsibility for the final content. All authors contributed to writing and revising the paper and agreed to submission.

## CONFLICT OF INTEREST STATEMENT

The authors declare that they have no conflict of interest.

### ETHICS STATEMENT

This study was approved by the Ethics Committee of Shanghai Guanghua Hospital of Integrated Traditional Chinese and Western Medicine and was in accordance with the 1964 Helsinki declaration and its later amendments or comparable ethical standards. Written informed consent to participate was obtained from all of the individual participants included in the study.

### TRIAL REGISTRATION

The trial was registered in Chinese Clinical Trial Registry (ChiCTR2000033778).

### PEER REVIEW

The peer review history for this article is available at https://publons.com/publon/10.1002/brb3.2913.

## Data Availability

The data sets used and analyzed during the current study are available from the corresponding author on reasonable request.

## References

[brb32913-bib-0001] American Society of Anesthesiologists Task Force on Acute Pain Management . (2012). Practice guidelines for acute pain management in the perioperative setting. Anesthesiologists, 116, 248–273. 10.1097/ALN.0b013e31823c1030 22227789

[brb32913-bib-0002] Boissoneault, J. , Penza, C. W. , George, S. Z. , Robinson, M. E. , & Bishop, M. D. (2020). Comparison of brain structure between pain‐susceptible and asymptomatic individuals following experimental induction of low back pain. Spine Journal, 20, 292–299. 10.1016/j.spinee.2019.08.015 PMC6995409 31479781

[brb32913-bib-0003] Chen, H. , Qi, G. , Zhang, Y. , Huang, Y. , Zhang, S. , Yang, D. , He, J. , Mu, L. , Zhou, L. , & Zeng, M. (2021). Altered dynamic amplitude of low‐frequency fluctuations in patients with migraine without aura. Frontiers in Human Neuroscience, 15, 636472. 33679354 10.3389/fnhum.2021.636472 PMC7928334

[brb32913-bib-0004] Chen, T. , Zhang, W. W. , Chu, Y. X. , & Wang, Y. Q. (2020). Acupuncture for pain management: Molecular mechanisms of action. American Journal of Chinese Medicine, 48, 793–811. 32420752 10.1142/S0192415X20500408

[brb32913-bib-0005] Chen, X. , Lu, B. , & Yan, C. (2018). Reproducibility of R‐fMRI metrics on the impact of different strategies for multiple comparison correction and sample sizes. Human Brain Mapping Journal, 39, 300–318. 10.1002/hbm.23843 PMC6866539 29024299

[brb32913-bib-0006] Coppes, O. J. M. , Yong, R. J. , Kaye, A. D. , & Urman, R. D. (2020). Patient and surgery‐related predictors of acute postoperative pain. Current Pain and Headache Reports, 24, 12. 32072315 10.1007/s11916-020-0844-3

[brb32913-bib-0007] Ge, X. , Wang, L. , Pan, L. , Ye, H. , Zhu, X. , Fan, S. , Feng, Q. , Yu, W. , & Ding, Z. (2022). Amplitude of low‐frequency fluctuation after a single‐trigger pain in patients with classical trigeminal neuralgia. The journal of headache and pain, 23, 117. 36076162 10.1186/s10194-022-01488-8 PMC9461270

[brb32913-bib-0008] Goffaux, P. , Girard‐Tremblay, L. , Marchand, S. , Daigle, K. , & Whittingstall, K. (2014). Individual differences in pain sensitivity vary as a function of precuneus reactivity. Brain Topography, 27, 366–374. 23636269 10.1007/s10548-013-0291-0

[brb32913-bib-0009] Hou, A. , Zheng, M. , Hua, X. , Huo, B. , & Shen, J. (2020). Electroacupuncture‐related metabolic brain connectivity in neuropathic pain due to brachial plexus avulsion injury in rats. Frontiers in Neural Circuits, 14, 1–11. 32625066 10.3389/fncir.2020.00035 PMC7313422

[brb32913-bib-0010] Hsia, H. L. , Takemoto, S. , Van De Ven, T. , Pyati, S. , Buchheit, T. , Ray, N. , Wellman, S. , Kuo, A. , Wallace, A. , & Raghunathan, K. (2018). Acute pain is associated with chronic opioid use after total knee arthroplasty. Regional Anesthesia and Pain Medicine, 43, 705–711. 29975257 10.1097/AAP.0000000000000831 PMC6319560

[brb32913-bib-0011] Huang, S. W. , Wang, W. T. , Chou, L. C. , Liao, C. D. , Liou, T. H. , & Lin, H. W. (2015). Osteoarthritis increases the risk of dementia: A nationwide cohort study in Taiwan. Scientific Reports, 5, 10145. 25984812 10.1038/srep10145 PMC4434986

[brb32913-bib-0012] Kang, B. , Ma, J. , Shen, J. , Xu, H. , Wang, H. , Zhao, C. , Xie, J. , Zhong, S. , Gao, C. , Xu, X. , A, X. , Xiao, L. , & Xu, J. (2022). Altered brain activity in end‐stage knee osteoarthritis revealed by resting‐state functional magnetic resonance imaging. Brain and Behavior, 12, e2479. 34967156 10.1002/brb3.2479 PMC8785636

[brb32913-bib-0013] Katherine, T. M. , Kenneth, A. W. I. , & Sean, C. M. (2019). Altered cervical spinal cord resting state activity in fibromyalgia. Arthritis & Rheumatology, 71, 441–450. 30281205 10.1002/art.40746 PMC6393192

[brb32913-bib-0014] Khoshnejad, M. , Roy, M. , Martinu, K. , Chen, J. I. , Cohen‐Adad, J. , Grondin, S. , & Rainville, P. (2017). Brain processing of the temporal dimension of acute pain in short‐term memory. Pain, 158, 2001–2011. 28817417 10.1097/j.pain.0000000000001003

[brb32913-bib-0015] Kim, D. , Chae, Y. , Park, H. J. , & Lee, I. S. (2021). Effects of chronic pain treatment on altered functional and metabolic activities in the brain: A systematic review and meta‐analysis of functional neuroimaging studies. Frontiers in Neuroscience, 15, 684926. 34290582 10.3389/fnins.2021.684926 PMC8287208

[brb32913-bib-0016] Kong, J. , Wang, Z. , Leiser, J. , Minicucci, D. , Edwards, R. , Kirsch, I. , Wasan, A. D. , Lang, C. , Gerber, J. , Yu, S. , Napadow, V. , Kaptchuk, T. J. , & Gollub, R. L. (2018). NeuroImage : Clinical enhancing treatment of osteoarthritis knee pain by boosting expectancy : A functional neuroimaging study. NeuroImage: Clinical, 18, 325–334. 29868449 10.1016/j.nicl.2018.01.021 PMC5984593

[brb32913-bib-0017] Li, C. , Pei, Q. , Chen, Y. , Luo, X. , Yang, N. , Li, T. T. , Ding, J. , & Wang, Y. (2020). The response‐time relationship and covariate effects of acupuncture for chronic pain: A systematic review and model‐based longitudinal meta‐analysis. European Journal of Pain (United Kingdom), 24, 1653–1665. 10.1002/ejp.1617 32533885

[brb32913-bib-0018] Liang, Q. , Zhang, K. , Wang, S. , Xu, X. , Liu, Y. , Cui, S. , & Liu, L. (2020). Acupuncture for cancer pain—An adjuvant therapy for cancer pain relief. American Journal of Chinese Medicine, 48, 1769–1786. 33300479 10.1142/S0192415X20500883

[brb32913-bib-0019] Liu, S. , Yin, N. , Ma, R. , Cao, H. , Jing, C. , Zhang, Y. , Chen, D. , Zhang, J. , Wu, Y. , Feng, J. , & Wu, J. (2020). Abnormal topological characteristics of brain white matter network relate to cognitive and emotional deficits of non‐small cell lung cancer (NSCLC) patients prior to chemotherapy. International Journal of Neuroscience, 132, 328–337. 33106073 10.1080/00207454.2020.1813130

[brb32913-bib-0020] Liu, Z. , Liu, Y. , Xu, H. , He, L. , Chen, Y. , Fu, L. , Li, N. , Lu, Y. , Su, T. , Sun, J. , Wang, J. , Yue, Z. , Zhang, W. , Zhao, J. , Zhou, Z. , Wu, J. , Zhou, K. , Ai, Y. , Zhou, J. , … Liu, B. (2017). Effect of electroacupuncture on urinary leakage among women with stress urinary incontinence: A randomized clinical trial. JAMA, 317, 2493–2501. 28655016 10.1001/jama.2017.7220 PMC5815072

[brb32913-bib-0021] Luo, J. , Yang, R. , Yang, W. , Duan, C. , Deng, Y. , Zhang, J. , Chen, J. , & Liu, J. (2020). Increased amplitude of low‐frequency fluctuation in right angular gyrus and left superior occipital gyrus negatively correlated with heroin use. Front Psychiatry, 11, 429. 32719620 10.3389/fpsyt.2020.00492 PMC7350776

[brb32913-bib-0022] Ma, M. , Zhang, H. , Liu, R. , Liu, H. , Yang, X. , Yin, X. , Chen, S. , & Wu, X. (2020). Static and dynamic changes of amplitude of low‐frequency fluctuations in cervical discogenic pain. Frontiers in Neuroscience, 14, 1–7. 32760245 10.3389/fnins.2020.00733 PMC7372087

[brb32913-bib-0023] Mawla, I. , Ichesco, E. , Zöllner, H. J. , Edden, R. A. E. , Chenevert, T. , Buchtel, H. , Bretz, M. D. , Sloan, H. , Kaplan, C. M. , Harte, S. E. , Mashour, G. A. , Clauw, D. J. , Napadow, V. , & Harris, R. E. (2021). Greater somatosensory afference with acupuncture increases primary somatosensory connectivity and alleviates fibromyalgia pain via insular γ‐aminobutyric acid: A randomized neuroimaging trial. Arthritis and Rheumatology, 73, 1318–1328. 33314799 10.1002/art.41620 PMC8197768

[brb32913-bib-0024] Michaelides, A. , & Zis, P. (2019). Depression, anxiety and acute pain: Links and management challenges. Postgraduate Medicine, 131, 438–444. 31482756 10.1080/00325481.2019.1663705

[brb32913-bib-0025] Morton, D. L. , Samdhu, J. S. , & Jones, A. K. (2016). Brain imagine of pain: State of the art. Journal of Pain Research, 9, 613–624. 27660488 10.2147/JPR.S60433 PMC5019436

[brb32913-bib-0026] Pereira‐Pedro, A. S. , & Bruner, E. (2016). Sulcal pattern, extension, and morphology of the precuneus in adult humans. Annals of Anatomy, 208, 85–93. 27210059 10.1016/j.aanat.2016.05.001

[brb32913-bib-0027] Price, D. D. (2000). Psychological and neural mechanisms of the affective dimension of pain. Science, 288, 1769–1772. 10846154 10.1126/science.288.5472.1769

[brb32913-bib-0028] Qi, C. X. , Huang, X. , & Shen, Y. (2018). Altered intrinsic brain activities in patients with diabetic retinopathy using amplitude of low‐frequency fluctuation: A resting‐state fMRI study. Diabetes, Metabolic Syndrome and Obesity: Targets and Therapy, 14, 251–257. 10.2147/DMSO.S259476 PMC7434521 32884311

[brb32913-bib-0029] Qiu, K. , Jing, M. , Sun, R. , Yang, J. , Liu, X. , He, Z. , Yin, S. , Lan, Y. , Cheng, S. , Gao, F. , Liang, F. , & Zeng, F. (2016). The status of the quality control in acupuncture‐neuroimaging studies. Evidence‐based Complementary and Alternative Medicine, 2016, 3685785. 27242911 10.1155/2016/3685785 PMC4875991

[brb32913-bib-0030] Schwedt, T. J. , Chiang, C. C. , Chong, C. D. , & Dodick, D. W. (2015). Functional MRI of migraine. The Lancet Neurology, 14, 81–91. 25496899 10.1016/S1474-4422(14)70193-0 PMC11318354

[brb32913-bib-0031] Seghier, M. L. (2013). The angular gyrus: Multiple functions and multiple subdivisions. The Neuroscientist, 19, 43–61. 22547530 10.1177/1073858412440596 PMC4107834

[brb32913-bib-0032] Small, C. , & Laycock, H. (2020). Acute postoperative pain management. British Journal of Surgery, 107, e70–e80. 31903595 10.1002/bjs.11477

[brb32913-bib-0033] Summers, S. , Mohile, N. , McNamara, C. , & Osman, B. (2020). Analgesia in total knee arthroplasty: Current pain control modalities and outcomes. The Journal of Bone and Joint Surgery, 102, 719–727. 31985507 10.2106/JBJS.19.01035

[brb32913-bib-0034] Szucs, D. , & Ioannidis, J. P. (2020). Sample size evolution in neuroimaging research: An evaluation of highly‐cited studies (1990–2012) and of latest practices (2017–2018) in high‐impact journals. Neuroimage, 221, 117164. 32679253 10.1016/j.neuroimage.2020.117164

[brb32913-bib-0035] Tedesco, D. , Gori, D. , Desai, K. R. , Asch, S. , Carroll, I. R. , Curtin, C. , McDonald, K. M. , Fantini, M. P. , & Hernandez‐Boussard, T. (2017). Drug‐free interventions to reduce pain OR opioid consumption after total knee arthroplasty a systematic review and meta‐analysis. JAMA Surgery, 152, 1–13. 10.1001/jamasurg.2017.2872 PMC5831469 28813550

[brb32913-bib-0036] Teng, C. , Zhou, J. , Ma, H. , Tan, Y. , Wu, X. , Guan, C. , Qiao, H. , Li, J. , Zhong, Y. , Wang, C. , & Zhang, N. (2018). Abnormal resting state activity of left middle occipital gyrus and its functional connectivity in female patients with major depressive disorder 17 psychology and cognitive sciences 1701 psychology. BMC Psychiatry [Electronic Resource], 18, 370. 30477561 10.1186/s12888-018-1955-9 PMC6258168

[brb32913-bib-0037] Tian, Z. , Guo, Y. , Yin, T. , Xiao, Q. , Ha, G. , Chen, J. , Wang, S. , Lan, L. , & Zeng, F. (2021). Acupuncture modulation effect on pain processing patterns in patients with migraine without aura. Frontiers in Neuroscience, 15, 729218. 34512254 10.3389/fnins.2021.729218 PMC8427167

[brb32913-bib-0038] Tu, Y. , Zeng, F. , Lan, L. , Li, Z. , Maleki, N. , Liu, B. , Chen, J. , Wang, C. , Park, J. , Lang, C. , Yujie, G. , Liu, M. , Fu, Z. , Zhang, Z. , Liang, F. , & Kong, J. (2020). An fMRI‐based neural marker for migraine without aura. Neurology, 94, e741–e751. 31964691 10.1212/WNL.0000000000008962 PMC7176301

[brb32913-bib-0039] Wen, Q. , Ma, P. , Xiaohui, D. , Sun, R. , Lan, L. , Yin, T. , Qu, Y. , Liu, Y. , Xiao, Q. , & Zeng, F. (2021). Neuroimaging studies of acupuncture on low back pain: Asystematic rewiew. Frontiers in Neuroscience, 20, 730322. 10.3389/fnins.2021.730322 PMC8488100 34616275

[brb32913-bib-0040] Xiao, L. Y. , Wang, X. R. , Yang, Y. , Yang, J. W. , Cao, Y. , Ma, S. M. , Li, T. R. , & Liu, C. Z. (2018). Applications of acupuncture therapy in modulating plasticity of central nervous system. Neuromodulation, 21, 762–776. 29111577 10.1111/ner.12724

[brb32913-bib-0041] Xu, T. , Zhang, Y. , Wang, C. , Liao, H. , Zhou, S. , Li, D. , Huang, S. , Shi, Y. , Wang, Z. , Chen, J. , Liang, F. R. , & Zhao, L. (2020). Brain structural and functional differences between pure menstrual migraine and menstrually‐related migraine. Scientific Reports, 10, 16454. 33020518 10.1038/s41598-020-73399-0 PMC7536204

[brb32913-bib-0042] Yan, C. G. , Cheung, B. , Kelly, C. , Colcombe, S. , Craddock, R. C. , Di Martino, A. , Li, Q. , Zuo, X. N. , Castellanos, F. X. , & Milham, M. P. (2013). A comprehensive assessment of regional variation in the impact of head micromovements on functional connectomics. Neuroimage, 76, 183–201. 23499792 10.1016/j.neuroimage.2013.03.004 PMC3896129

[brb32913-bib-0043] Yang, J. , Wahner‐Roedler, D. L. , Zhou, X. , Johnson, L. A. , Do, A. , Pachman, D. R. , Chon, T. Y. , Salinas, M. , Millstine, D. , & Bauer, B. A. (2021). Acupuncture for palliative cancer pain management: Systematic review. BMJ Supportive and Palliative Care, 11, 264–270. 10.1136/bmjspcare-2020-002638 PMC8380897 33441387

[brb32913-bib-0044] Yang, L. , Yan, Y. , Li, Y. , Hu, X. , Lu, J. , Chan, P. , Yan, T. , & Han, Y. (2020). Frequency‐dependent changes in fractional amplitude of low‐frequency oscillations in Alzheimer's disease: A resting‐state fMRI study. Brain Imaging and Behavior, 14, 2187–2201. 31478145 10.1007/s11682-019-00169-6

[brb32913-bib-0045] Yu, S. , Ortiz, A. , Gollub, R. L. , Wilson, G. , Gerber, J. , Park, J. , Huang, Y. , Shen, W. , Chan, S.‐T. , Wasan, A. D. , Edwards, R. R. , Napadow, V. , Kaptchuk, T. J. , Rosen, B. , & Kong, J. (2020). Acupuncture treatment modulates the connectivity of key regions of the descending pain modulation and reward systems in patients with chronic low back pain. Journal of Clinical Medicine, 9, 1719. 32503194 10.3390/jcm9061719 PMC7356178

[brb32913-bib-0046] Zang, Y. F. , He, Y. , Zhu, C. Z. , Cao, Q. J. , Sui, M. Q. , Liang, M. , Tian, L. X. , Jiang, T. Z. , & Wang, Y. F. (2007). Altered baseline brain activity in children with ADHD revealed by resting‐state functional MRI. Brain and Development, 29, 83–91. 16919409 10.1016/j.braindev.2006.07.002

[brb32913-bib-0047] Zhang, B. , Jung, M. , Tu, Y. , Gollub, R. , Lang, C. , Ortiz, A. , Park, J. , Wilson, G. , Gerber, J. , Mawla, I. , Chan, S. T. , Wasan, A. , Edwards, R. , Lee, J. , Napadow, V. , Kaptchuk, T. , Rosen, B. , & Kong, J. (2019). Identifying brain regions associated with the neuropathology of chronic low back pain: A resting‐state amplitude of low‐frequency fluctuation study. British Journal of Anaesthesia, 123, e303–e311. 30948036 10.1016/j.bja.2019.02.021 PMC6676015

[brb32913-bib-0048] Zhao, J. , & Davis, S. P. (2019). An integrative review of multimodal pain management on patient recovery after total hip and knee arthroplasty. International Journal of Nursing Studies, 98, 94–106. 31352132 10.1016/j.ijnurstu.2019.06.010

[brb32913-bib-0049] Zhong, S. , Huang, H. , Xie, J. , Zhao, L. , Song, X. L. , Chen, Y. L. , & Xiao, L. B. (2019). Application of electroacupuncture for postoperative pain management after total knee arthroplasty: A study protocol for a single‐blinded, randomised placebo‐controlled trial. BMJ Open, 9, 1–6. 10.1136/bmjopen-2018-026084 PMC6500353 30962235

